# Fear learning circuitry is biased toward generalization of fear associations in posttraumatic stress disorder

**DOI:** 10.1038/tp.2015.196

**Published:** 2015-12-15

**Authors:** R A Morey, J E Dunsmoor, C C Haswell, V M Brown, A Vora, J Weiner, D Stjepanovic, H R Wagner, VA Mid-Atlantic MIRECC Workgroup, Mira Brancu, Christine E Marx, Jennifer C Naylor, Elizabeth Van Voorhees, Katherine H Taber, Jean C Beckham, Patrick S Calhoun, John A Fairbank, Steven T Szabo, K S LaBar

**Affiliations:** 1 Mid-Atlantic Mental Illness Research Education and Clinical Center, Durham VA Medical Center, Durham, NC, USA; 2 Department of Psychiatry and Behavioral Sciences, Duke University, Durham, NC, USA; 3 Duke-UNC Brain Imaging and Analysis Center, Duke University, Durham, NC, USA; 4 Department of Psychology and Neuroscience, Center for Cognitive Neuroscience, Duke University, Durham, NC, USA; 5 Department of Psychology, New York University, New York, NY, USA; 6 Department of Psychology, Virginia Polytechnic Institute and State University, Blacksburg, VA, USA; 7 Virginia Tech-Carilion Research Institute, Virginia Polytechnic Institute and State University, Roanoke, VA, USA; 8 School of Management, University of Iowa, Iowa City, IA, USA

## Abstract

Fear conditioning is an established model for investigating posttraumatic stress disorder (PTSD). However, symptom triggers may vaguely resemble the initial traumatic event, differing on a variety of sensory and affective dimensions. We extended the fear-conditioning model to assess generalization of conditioned fear on fear processing neurocircuitry in PTSD. Military veterans (*n*=67) consisting of PTSD (*n*=32) and trauma-exposed comparison (*n*=35) groups underwent functional magnetic resonance imaging during fear conditioning to a low fear-expressing face while a neutral face was explicitly unreinforced. Stimuli that varied along a neutral-to-fearful continuum were presented before conditioning to assess baseline responses, and after conditioning to assess experience-dependent changes in neural activity. Compared with trauma-exposed controls, PTSD patients exhibited greater post-study memory distortion of the fear-conditioned stimulus toward the stimulus expressing the highest fear intensity. PTSD patients exhibited biased neural activation toward high-intensity stimuli in fusiform gyrus (*P*<0.02), insula (*P*<0.001), primary visual cortex (*P*<0.05), locus coeruleus (*P*<0.04), thalamus (*P*<0.01), and at the trend level in inferior frontal gyrus (*P*=0.07). All regions except fusiform were moderated by childhood trauma. Amygdala–calcarine (*P*=0.01) and amygdala–thalamus (*P*=0.06) functional connectivity selectively increased in PTSD patients for high-intensity stimuli after conditioning. In contrast, amygdala–ventromedial prefrontal cortex (*P*=0.04) connectivity selectively increased in trauma-exposed controls compared with PTSD patients for low-intensity stimuli after conditioning, representing safety learning. In summary, fear generalization in PTSD is biased toward stimuli with higher emotional intensity than the original conditioned-fear stimulus. Functional brain differences provide a putative neurobiological model for fear generalization whereby PTSD symptoms are triggered by threat cues that merely resemble the index trauma.

## Introduction

Individuals with posttraumatic stress disorder (PTSD) exhibit anxiety-related behaviors based on reminders of past trauma, have difficulty extinguishing fear associations, and display frequent reawakening of fear associations.^1^ They also generalize fear and anxiety elicited by traumatic events to a variety of triggers that resemble the initial trauma.^2, 3^ Fear conditioning is a widely studied model of avoidance and re-experiencing symptoms of PTSD.^4^ Patients with PTSD often show differences in fear acquisition and extinction relative to trauma victims without PTSD.^4^ However, symptom triggers may only vaguely resemble the index trauma and may differ from the trauma experience in shape, context, emotional valence, smell, semantic association, and other dimensions.^5^ Indeed, DSM-5 criterion B specifies symptoms of ‘intense psychological distress and physiological reactivity may symbolize or resemble the traumatic events.' Extant fear conditioning models are limited by repeated use of the same cue to trigger the initial trauma. Although fear conditioning and extinction in PTSD have received widespread attention,^6^ fear generalization in PTSD has not been well-studied,^7^ particularly with neuroimaging. Our goal was to extend the fear-conditioning model to assess generalization of conditioned fear on fear processing neurocircuitry and behavior in PTSD.

**Figure 1:**
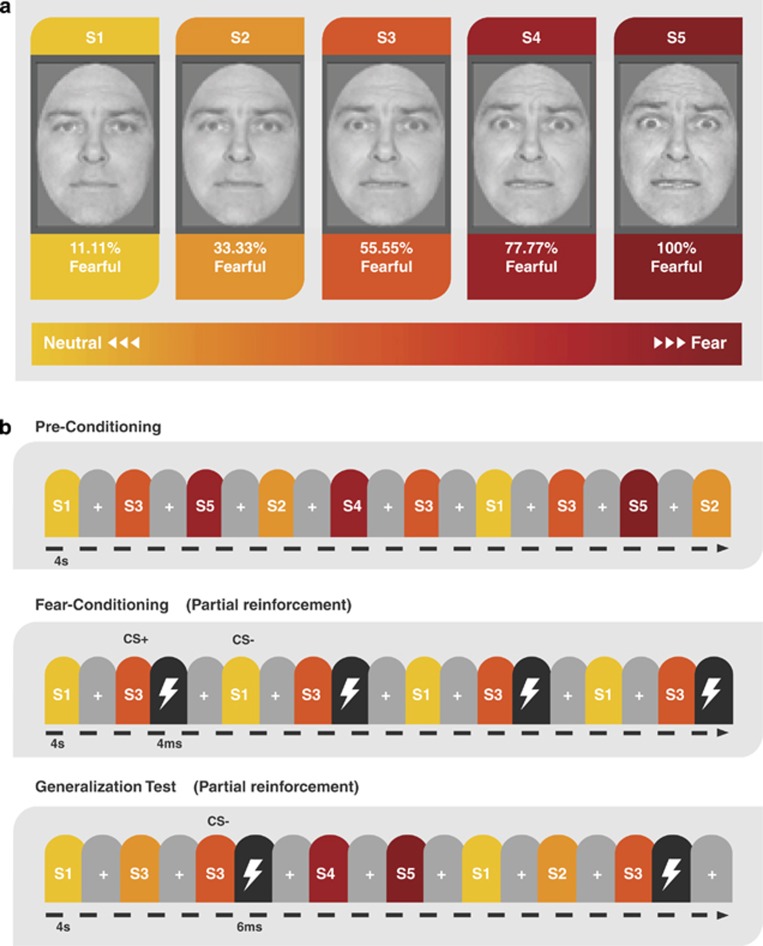
Stimuli and task design. (**a**) Generalization was assessed with images of five facial morphs of the same identity that ranged from neutral-to-fearful end points. (**b**) During preconditioning, participants were exposed to all the five facial morphs before fear conditioning to assess the baseline behavioral and neural responses. Fear learning was accomplished in two runs by pairing an electrical shock (US) with presentation of the S3 morph (CS+) on 6 out of 18 trials (33%), whereas S1 was never paired with shock (CS−). Morphs S2, S4 and S5 were not presented during fear conditioning. During four runs of the generalization test, all the five morphs (S1–S5) were presented and S3 was intermittently reinforced with a shock (4 of 12 trials; 33%). US, unconditioned stimulus.

We adopted a validated fear-conditioning paradigm that incorporates faces of the same identity expressing various intensities of fear (Figure 1). We have previously shown that healthy subjects tend to generalize conditioned fear towards faces that resemble a conditioned stimulus (CS) but express higher emotional intensity.^8, 9^ Similarly, animal conditioning studies have revealed asymmetrical generalization towards unreinforced stimuli of higher physical intensity than a CS along dimensions of loudness or brightness, an effect referred to as intensity generalization.^10^ Here, we extend the concept of intensity generalization to examine whether PTSD patients are sensitive to the emotional intensity of unreinforced stimuli after an aversive experience with a stimulus of moderate intensity. Our goal was to investigate experience-dependent changes in fear neurocircuitry, behavior, memory, and correlations with lifetime trauma exposure and clinical symptoms before and after fear conditioning in PTSD as compared with trauma-exposed veterans without PTSD.

Conditioning paradigms using fear-relevant stimuli, and testing for generalization gradient asymmetries as a function of CS intensity, serve as an appropriate model for PTSD, given that these stimuli exhibit resistance to extinction learning and that many symptom triggers involve stimuli with some inherent threat value.^11^ Critically, neural activity to each stimulus was obtained pre- and post-fear conditioning with functional magnetic resonance imaging (fMRI) to observe experience-dependent changes resulting from fear conditioning. This pre–post training design is commonly used in animal neurophysiology to examine representational plasticity in sensory cortex following Pavlovian fear conditioning^12^ and, in the current study, to control for potential baseline differences in neural response between PTSD and non-PTSD controls.^11^

We hypothesized that PTSD patients would exhibit robust experience-dependent changes in fear neurocircuitry, and retrospective memory biases for faces that expressed higher fear intensity, as compared with trauma-exposed control subjects. Predictions about the neurocircuitry mediating fear generalization were based on cross-species evidence of brain systems involved in acquiring and expressing learned fear,^13^ as well as fMRI investigations of fear generalization in healthy adults^9, 14^ and patients with generalized anxiety disorder.^15, 16^ Specifically, we expected PTSD patients to exhibit enhanced generalization of neural activity following fear conditioning in regions of the ‘central autonomic-interoceptive network' commonly identified in fMRI investigations of human fear conditioning.^17^ This network includes the thalamus, based on its role in sensory integration of information for the amygdala during fear learning,^18^ and the striatum, based on its role in continuously updating the amygdala with expectancy information based on aversive prediction errors.^19^ Likewise, the dorsal anterior cingulate cortex is a part of key neurocircuitry involved in appraisal and expression of learned fear.^20^ Asymmetric stimulus-intensity generalization of neural activity was also predicted along the ventral visual stream, including the fusiform gyrus, via sensory enhancement of visual representations by the amygdala and the thalamus.^21^ Finally, the locus coeruleus is the site of synthesis of noradrenergic neurotransmitters released in response to acute stress or threat, and activity in this region has been linked to stress-induced overgeneralization of memory representations.^22^

We hypothesized that each of these regions would show greater pre- to post-fear conditioning increases in neural activity in PTSD patients compared with trauma-exposed controls for stimuli of higher intensity than a learned threat (CS+). We also hypothesized that fear generalization in PTSD would be associated with changes in functional connectivity with the amygdala, given its central role in conditioning-induced changes in brain plasticity. Finally, we predicted that childhood trauma exposure would enhance generalization biases in fear neurocircuitry, in line with prior evidence that lifetime trauma exposure is strongly correlated with PTSD severity.^23^

## Materials and methods

### Participants

The participants (*n*=67) were recruited from February 2011 through April 2014, from a repository^24^ of 3500 US military veterans. All the participants served since 11 September 2001, and most were deployed to Iraq and/or Afghanistan military conflicts. All the participants provided written informed consent to participate in procedures approved by the Institutional Review Boards at Duke University Medical Center and Durham VA Medical Center. Participants underwent screening for inclusion in the study and subsequent clinical assessment of PTSD symptoms, trauma exposure, psychiatric comorbidities and medication use described in the Supplementary Information. Participants' demographic and clinical features (Supplementary Table 1) were matched for age, sex and race, and maternal education as a proxy for intelligence (Intelligence Quotient). The PTSD group had greater childhood trauma, combat exposure, depressive symptoms, alcohol use and psychotropic medication usage that were controlled in our fMRI analyses.

### Stimuli and paradigm

The experimental paradigm, based on Dunsmoor *et al.*,^9^ consisted of three consecutive stages that occurred in the same order for each participant: preconditioning, fear conditioning and generalization. All five face stimuli were presented during preconditioning to measure baseline neural responses (Figure 1). Subjects rated the intensity of facial expressions on each trial during preconditioning. Fear conditioning involved presentation of a face depicting a subtle fear (CS+, 55% fear) that predicted the occurrence of a mildly aversive electric shock US (unconditioned stimulus) on 6 out of 18 CS+ trials (33%), intermixed with an unpaired face depicting minimal fear (CS−, 11% fear). A reinforcement rate of 33% is sufficient to induce differential fear conditioning between the CS+ and CS−.^25^ Partial reinforcement is often used in fMRI studies of human fear conditioning to prevent rapid extinction,^26^ which is important in the current study for generalizing to stimuli not previously paired with shock. The generalization stimuli (S2, 33% fear; S4, 77% fear; S5, 99% fear) were gradations of a single facial identity morphed incrementally between neutral and fearful end points (Figure 1a).^27^ During the generalization test, the CS+ was intermittently paired with the US in 4 out of 12 trials (33%) to offset the effects of extinction over the extended testing session (steady-state generalization test).^28^ Stimulus duration was 4 s, and subjects were not informed of any CS–US contingencies (Supplementary Information). During fear conditioning and generalization, subjects rated expectancy for receiving a shock on each trial to assess fear-conditioning success. Activation from preconditioning baseline was subtracted from conditioned-fear generalization to extract learning-induced changes in responsivity. Usable skin conductance response data were available in only 30% of subjects owing to technical challenges with filtering noise in the MRI environment, and is, therefore, not reported. Thus, fear-conditioning success was assessed with trial-by-trial expectancy ratings, which is considered a valid measure of fear conditioning with strong face-, diagnostic-, predictive- and construct-validity.^29^ At the conclusion of the scan, in a surprise post-generalization retrospective memory test for recognition of the CS+, subjects chose the ‘correct' CS+ from among the five morph values presented in a single montage that was arranged in a random order (Supplementary Information). Subjects were permitted to select multiple faces if they believed more than one face was associated with shock.

### fMRI acquisition and data analysis

Structural and functional MRI data were acquired and preprocessed as detailed in the Supplementary Information. The overall approach for the analysis of fMRI data consisted of four main steps: (i) the hypothesis-generating step identified functionally defined regions of interest (ROIs) from a whole-brain contrast of CS+>CS− during fear conditioning, with FSL whole-brain correction for multiple comparisons; (ii) activation in these ROIs was interrogated in the hypothesis-testing step to assess response to the five facial morphs presented in the fear-generalization stage; (iii) activation in these ROIs to the facial morphs was interrogated from the preconditioning stage and subtracted from the generalization response; and (iv) the difference in activation for the facial morphs expressing greater fear (S4, S5) and lesser fear (S2) than the CS+ were compared with the CS− as a control stimulus to assess activation associated with generalization. The functional ROIs were consistent with our prior work in a nonclinical sample.^9^ In a separate analysis to assess generalization to safety-signal learning, all the preceding steps were duplicated, except that ROIs in the hypothesis-generating step were obtained by contrasting CS−>CS+, and the final step compared facial morphs with the CS+ condition, given that the analysis was targeted to identify brain regions whose activity signaled safety learning (Supplementary Information).

Between-group analyses involved both voxel-based and ROI-based statistics (*t*-tests/analyses of variance/analyses of covariance and planned comparisons using the individual subjects' activation *z*-maps and mean percent-signal-change from functional ROIs). Hypothesis testing was conducted in each ROI with a 2 × 3 × 2 repeated-measures design that included diagnosis, stimulus-intensity and time (image volumes). The diagnosis × stimulus-intensity interaction, reflecting generalization differences in the PTSD compared with control group, was the key outcome of interest (Supplementary Information). All the tests included regressors for alcohol use, depression, childhood trauma, combat exposure and dummy variables that coded for treatment with antidepressant, mood stabilizer, antipsychotic and benzodiazepine medication, as described in our earlier work.^30^

### Amygdala functional connectivity analysis

The goal of the connectivity analysis was to measure task-modulated functional connections between the amygdala and brain regions associated with generalization of learned fear. We adapted the generalized psychophysiological interaction analysis that provides improved model fit compared with PPI.^31^ Functional connectivity was calculated for each subject between anatomically defined left and right amygdala seeds and target regions that included fusiform gyrus, thalamus, ventromedial prefrontal cortex (vmPFC) and primary visual cortex as defined in the hypothesis-generating step (Supplementary Information).

## Results

### Baseline results

Baseline ratings before acquisition (preconditioning) revealed a (Supplementary Figure 1a) significant main effect of stimulus-intensity (F_9,53_=351.25; *P*<0.0001), but no effect of diagnosis (F_1,61_=0.01; *P*>0.9), nor a stimulus-intensity × diagnosis interaction (F_9,53_=0.62; *P*>0.6). Repeated-measures analysis of variance with diagnosis as the between-group factor (PTSD, control) and stimulus-intensity with low (S1, S2) or high (S4, S5) intensity as within-group factors (Supplementary Figure 1b) found that no ROI showed a main effect of diagnosis (*P*-values >0.2). There was a significant stimulus-intensity × diagnosis interaction in the right fusiform (F_1,57_=10.89; *P*=0.002), and at trend level in the right thalamus (F_1,57_=3.68; *P*<0.06). However, planned comparisons of the fusiform revealed that high-intensity stimuli had lower activation in the PTSD group than the trauma-exposed control group. The remaining ROIs (R-amygdala, R-calcarine, R-IFG, R-insula, locus coeruleus, L-thalamus) showed no stimulus-intensity × diagnosis interaction (*P*-values >0.2). These results established that there was no bias before fear conditioning toward high-intensity stimuli in the PTSD group (Supplementary Figure 1; Supplementary Table 2). Nevertheless, baseline activation for each face stimulus was subtracted from the activation to the same stimulus during generalization to adjust for any individual differences not evident at the group level. This approach is in keeping with neurophysiological investigations examining experience-dependent changes in neural activity from pre- to post-fear conditioning.^12^

### Fear learning-related results

Shock expectancy ratings were significantly higher for the CS+ than the CS− during fear-acquisition runs (F_1,58_=107.0; *P*<0.0001), indicating successful fear learning.^29^ There was no difference between groups (F_1,58_=0.19; *P*<0.67) or stimulus-type × group interaction (F_1,58_=0.002; *P*<0.96). Specifically, there were no between-group differences for S1 (F_1,58_=0.03; *P*=0.87) or S3 (F_1,58_=0.08; *P*>0.78) during conditioning. (Supplementary Figure 2).

Across the entire sample (PTSD and controls combined), fear learning-related activation (CS+>CS−) was found in primary visual cortex (calcarine), inferior frontal gyrus (IFG), insula, locus coeruleus and thalamus (Supplementary Figure 3; Supplementary Table 3). The neural learning response in the amygdala was correlated with the CS+ versus CS− and the S5 activations among the PTSD group (R=0.37; see Supplementary Figure 4) and in the entire group (R=0.35). In contrast, safety-learning activation (CS−>CS+) was found in the vmPFC and several other regions (Supplementary Table 3).

### Fear generalization-related results

**Figure 2:**
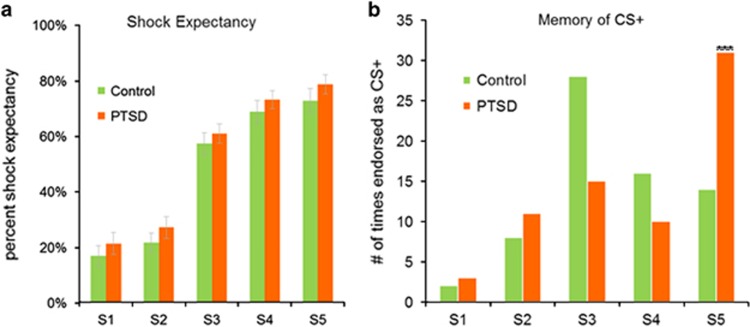
Shock expectancy and post-generalization memory of fear association. (**a**) Subjects provided ratings during the generalization task indicating the expectation of receiving a shock with each stimulus (S1–S5). There was no significant difference in shock expectancy between the PTSD and control groups (F_9,53_=1.5; *P*>0.2) nor any fear-level × diagnosis interaction (F_9,53_=0.47; *P*>0.7). As expected, there was a strong main effect of stimulus-intensity (F_9,53_=38.25; *P<*0.0001). Error bars indicate standard error of the mean. (**b**) The memory of fear association exhibits generalization in PTSD with a bias toward the face expressing the greatest fear (S5). The PTSD group misidentified the S5 stimulus as the CS+ (*χ*^2^_1_=10.19; *P*=0.001) more frequently (count=31; 45.2%) than the control group (count=14; 20.6%). The control group correctly identified the S3 as CS+ (count=28; 41.2%) more frequently than the PTSD group (count=15; 21.4%). There were no between-group differences for S4 (*χ*^2^_1_=0.09; *P*=0.76) or S2 (*χ^2^*_1_=2.86; *P*=0.10). The y axis represents count data (two per subject) and therefore does not have standard error bars. CS, conditioned stimulus; PTSD, posttraumatic stress disorder.

Shock expectancy ratings during generalization (Figure 2a) exhibited a main effect of fear level (F_9,53_=38.25; *P*<0.0001), demonstrating generalization of shock expectancy ratings from the CS+ to the faces that expressed higher-intensity stimuli (S4, S5). However, there was no effect of diagnosis (F_9,53_=1.5; *P*>0.2), nor a stimulus-intensity × diagnosis interaction (F_9,53_=0.47; *P*>0.7). Specifically, there were no between-group differences during generalization for the S1 (F_1,59_=0.64; *P*=0.43) and S3 (F_1,59_=0.16; *P*=0.69).

**Figure 3:**
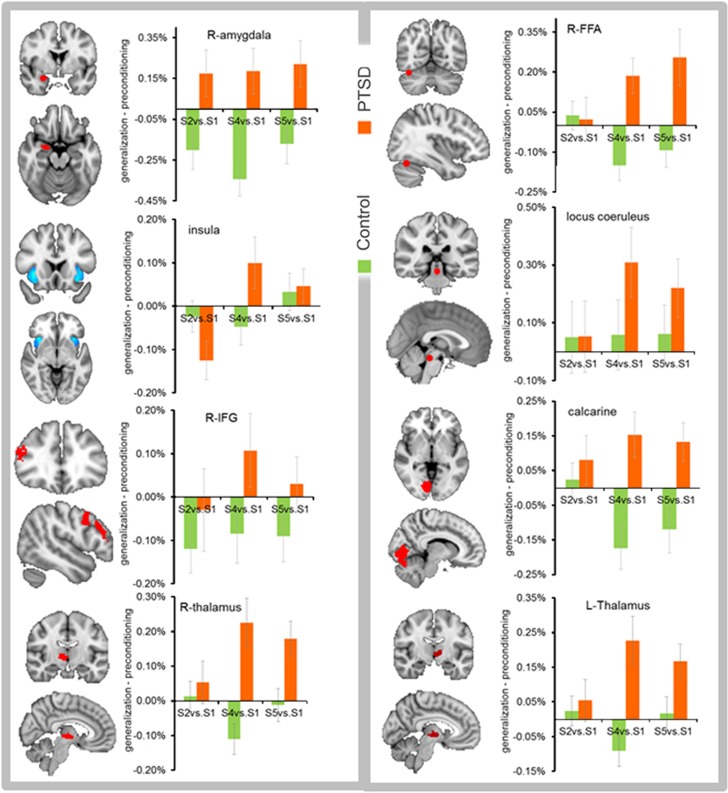
Regions of fear generalization bias in PTSD. Fear generalization response was biased toward higher emotional intensity than the original conditioned stimulus in R-fusiform (*P*<0.02), R-insula (*P*<0.001), locus coeruleus (*P*<0.04), L-thalamus (*P*<0.01), R-thalamus (*P*<0.005), R-primary visual cortex (calcarine; *P*<0.05) and at the trend level in R-IFG (*P*=0.07). Generalization bias was not observed in the amygdala, but the R-amygdala exhibited an overall increase in activation in the PTSD group for all stimulus intensities (*P*<0.0001). Error bars indicate standard error of the mean. IFG, inferior frontal gyrus; L, left; PTSD, posttraumatic stress disorder; R, right.

Baseline corrected ROI activation results relative to the S1 response (Figure 3; Table 1) showed a significant interaction of diagnosis × stimulus-intensity in the calcarine, fusiform, insula, locus coeruleus, thalamus and a trend-level effect in IFG. Overall, the PTSD group demonstrated a stronger response than the trauma-exposed control group toward higher-intensity stimuli (S4, S5) than the CS+. Specifically, planned comparisons showed higher activation in the PTSD group than the trauma-exposed control group for S2 in the amygdala; for S4 in the locus coeruleus, thalamus, calcarine, fusiform, and amygdala (trend level); and for S5 in the amygdala (trend level), calcarine and fusiform (trend level; Supplementary Table 4). The PTSD group had greater activation than the control group (main effect) in the amygdala, calcarine, IFG, thalamus and insula (trend level). A significant main effect of stimulus intensity was observed in the calcarine, IFG, insula, locus coeruleus and thalamus (Table 1). ROI activation results for all stimuli (S1–S5) without baseline correction (Supplementary Figure 5) and with baseline correction (Supplementary Figure 6) are included for reference.

Childhood trauma interacted with stimulus intensity to modulate regional activation in the calcarine, IFG, insula (trend level), locus coeruleus and thalamus. Combat exposure interacted with stimulus intensity to influence regional activation in the calcarine, locus coeruleus and fusiform (trend level). The medication covariates were found to have nonsignificant interactions with stimulus intensity (*P*-values >0.15), except antidepressant effects on activation in the fusiform and calcarine at trend level (Table 1).

### Functional connectivity results

**Figure 4:**
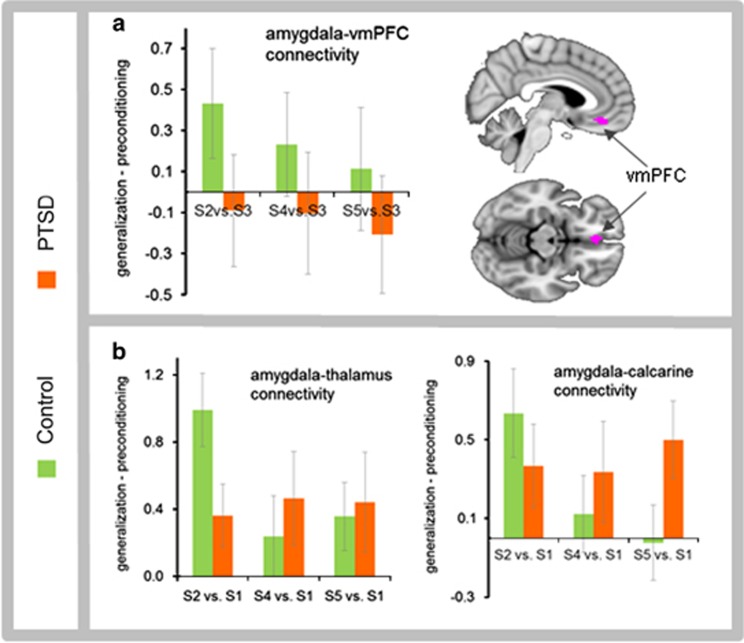
Task-modulated functional connectivity. (**a**) Task-modulated functional connectivity between the right amygdala and vmPFC (ROI obtained from CS−>CS+) showed a significant diagnosis × fear-level interaction (F(1,65)=4.22; *P*=0.04) suggestive of stronger connectivity in the trauma-exposed control group that was biased toward safety-signal learning. (**b**) Task-modulated functional connectivity between the right amygdala and thalamus showed trend-level diagnosis × fear-level interaction (F(1,65)=3.54; *P*=0.06), while connectivity between the right amygdala and the calcarine cortex showed a significant diagnosis × fear-level interaction (F(1,65)=6.35; *P*=0.01). Error bars indicate standard error of the mean. CS, conditioned stimulus; PTSD, posttraumatic stress disorder; ROI, region of interest; vmPFC, ventromedial prefrontal cortex.

Task-modulated functional connectivity between the right amygdala and calcarine cortex showed a significant diagnosis × stimulus-intensity interaction (F_1,65_=6.35; *P*=0.01), with a similar trend between the right amygdala and the thalamus (F_1,65_=3.54; *P*=0.06), indicating greater connectivity in the PTSD group to faces expressing higher stimulus intensity (S4, S5; Figure 4; Supplementary Table 5). Task-based connectivity between the right amygdala and fusiform was nonsignificant (*P*=0.16). Task-modulated connectivity between the right amygdala and vmPFC showed a significant diagnosis × stimulus-intensity interaction (F_1,65_=4.22; *P*=0.04), with greater connectivity in the control group to the lowest stimulus intensity (S2) that most closely resembled the CS− (safety signal). Functional connectivity between the left amygdala and all the target regions was nonsignificant.

### Post-generalization memory results

In the post-study recognition memory test, PTSD patients misidentified the face with the highest intensity stimulus (S5) as the CS+ (Figure 2b) more frequently than the control group (*χ*^2^_1_=10.19; *P*<0.001). S5 was selected most often by PTSD patients (count=31; 45.2%), indicating a strong memory bias to the face expressing the most fear, whereas controls selected the correct stimulus (S3) most often (count=28; 41.2%). There was no significant difference between the percentage of patients and controls who misidentified the other generalized stimuli (S4 (*χ*^2^_1_=0.09; *P*=0.76); S2 (*χ*^2^_1_=2.86; *P*=0.10)).

## Discussion

This study investigated generalization biases in fear neurocircuitry in military veterans with PTSD. Activity in a number of brain regions traditionally implicated in associative fear learning exhibited biased generalization towards stimuli of high emotional intensity after, but not before, fear conditioning in PTSD patients relative to controls. Activity in the amygdala broadly generalized to all stimuli in PTSD patients, and task-modulated functional connectivity of the amygdala with primary visual cortex and thalamus was biased in PTSD toward faces expressing high fear. Controls exhibited amygdala–vmPFC connectivity to a low-intensity stimulus that resembled the CS−, representing generalization of safety learning, whereas PTSD patients failed to exhibit similar patterns of amygdala–vmPFC connectivity. Neuroimaging results in PTSD were accompanied by a retrospective memory bias to falsely identify the highest intensity stimulus as the CS+. Collectively, these results provide new insights into how veterans with PTSD generalize fear from a single fear-learning episode to cues that have higher-intensity threat values than the original stimulus.

Brain regions showing intensity-based generalization in PTSD included areas along the ventral visual stream, such as primary visual cortex and fusiform gyrus, as well as sensory-integration components such as the thalamus, which have strong connections with the amygdala.^32^ Our functional connectivity results showed enhanced calcarine–amygdala and thalamo–amygdala functional coupling during fear generalization in PTSD. Fear generalization in PTSD was also facilitated by the locus coeruleus, which supports physiological and attentional components of the fight-or-flight response elicited by aversively conditioned stimuli through norepinephrine release.^33^ Finally, fear generalization in PTSD selectively recruited IFG and insula, which are part of a ventrolateral PFC circuit that integrates limbic responses with goal-directed actions, including holding affective material in working memory, directing attention to affectively salient information, and integrating somatic responses with decision-making processes.^34, 35^ In contrast to regions showing selective fear generalization, the amygdala exhibited a broader generalization gradient in PTSD, including enhanced responses to faces with low-threat values. Given that the amygdala activation showed a broader generalization pattern than its functional connectivity, additional neural interactions unidentified in the present analysis must constrain the pattern of functional connectivity with calcarine cortex and thalamus.

Our finding that controls exhibited a strong amygdala–vmPFC connectivity bias towards generalized cues that resemble the safety signal, whereas PTSD patients did not, builds on previous reports of behavioral deficits in safety learning^36^ and vmPFC disruption in extinction recall^6^ in PTSD. Given that extinction is widely considered a form of new inhibitory learning rather than erasing an established fear memory,^37^ shared neural mechanisms have been proposed for safety-signal learning and extinction learning.^38^ Combat veterans with PTSD lack effective safety signal learning as seen by poor modulation of fear in response to safety cues.^39^ Our connectivity results between vmPFC and amygdala are noteworthy in light of the essential role that vmPFC has in extinction, recall of learned extinction,^26^ and safety-signal learning through inhibition of the amygdala.^26^ Further research will be needed to explore amygdala–vmPFC connectivity associated with discriminating the safety signal from perceptually related stimuli, which is an important feature of resilience.^3^

The PTSD patients also exhibited a retrospective memory bias to falsely identify the face expressing the highest fear value as the CS+. Thus, evidence of group differences in generalization was found at the neural level and, with retrospective memory, there was no evidence for group differences in behavioral generalization assessed with shock expectancy. The lack of group differences in expectancy ratings during the fear-acquisition stage provides evidence that associative fear learning remains intact in PTSD. Likewise, the behavioral ratings of faces, as well as the neural activity during preconditioning, suggests that perceptual discrimination of emotion is also intact in PTSD. The apparent inconsistency in behavioral and neural findings could be explained by a lack of sensitivity of the behavioral measures to underlying neural mechanisms working to produce generalization. Indeed, these changes do not manifest at the behavioral level until later in the experiment, but only by probing a related cognitive construct that assessed retrospective memory for the CS+. This memory distortion is consistent with a co-variation bias prevalent in anxiety disorders.^40^ One possible mechanism is that enhanced fMRI activity to high threat-value faces alters memory consolidation in PTSD to yield a retrospective memory bias. Indeed, diagnostic criteria for PTSD includes an inability to recall key features of the traumatic event, whereas clinical evidence often describes delayed recall^41^ or a vivid central memory of the trauma that is strongly colored by emotional and sensory impressions.^42^ We previously reported that patients with PTSD rely on gist memory for the recognition of similar content across trauma-related images.^43^ Importantly, as baseline fear-expression ratings were matched across groups, we can rule out perceptual bias as an explanation for the memory differences. Future studies are warranted to determine whether the memory biases in PTSD are reversible and to identify neural mechanisms that contribute to this memory bias.

Our results indicate that exposure to childhood trauma predicted activation in the calcarine, IFG, insula, locus coeruleus and thalamus. It is well established that childhood maltreatment is associated with childhood poverty,^44^ which in turn is linked to aberrant functional connectivity in adulthood.^45^ Interestingly, both the groups in our sample experienced high rates of childhood trauma, albeit significantly higher in the PTSD group. It is well known that mild-to-moderate stress in childhood is required for healthy brain development, whereas more extreme low-stress and high-stress environments have negative consequences.^46, 47^ To the extent that maltreatment is a form of stress, it is possible that mild exposure could result in greater resilience in adulthood. We found that childhood trauma predicted the generalization response in components of the fear neurocircuitry. These findings provide neurobiological support for findings from many studies, including a large sample (*n*=2181) of individuals who experienced multiple violent assaults in childhood resulting in greater likelihood of PTSD following trauma in adulthood.^23^ Our results extend prior evidence that exposure to child abuse is a major environmental factor, which further interacts with genetic factors that contribute significant risk for severity and chronicity of adult PTSD.^48^

Although some clinical and experimental evidence suggests that PTSD patients are hyper-responsive to cues that portend threat, such as fearful faces,^11^ there was no evidence of baseline differences in neural activity (including the amygdala) or subjective ratings of emotional intensity in the present sample (Supplementary Figure 1b), consistent with our prior neuroimaging result using this paradigm in healthy adults.^9^ Neuroimaging research on amygdala responses to emotional versus affectively neutral faces in healthy adults is mixed, with earlier studies showing stronger amygdala responses^49^ but more recent studies showing equivalent amygdala activity to faces regardless of emotional expression.^50, 51, 52^ The lack of differential amygdala engagement before fear conditioning in PTSD versus controls is noteworthy given prior fMRI findings.^11, 53^ A critical distinction between this and prior studies in PTSD patients, however, is the use of an event-related design and repeated presentation of the same actor identity, as opposed to a block design and multiple face identities.^53^

We used fearful faces because prior research has demonstrated that fearful facial expressions tend to potentiate conditioned-fear responses, retard extinction learning and serve as a threat signal.^54^ These characteristics are salient for fear generalization in PTSD, thus enhancing ecological validity to the conditioning paradigm. Also, while the stimulus generalization literature has historically focused on perceptual similarity,^28^ intensity-based generalization^10^ provides unique insight into asymmetrical forms of fear generalization characteristic of anxiety and trauma disorders.^55^ Specifically, a number of cues reminiscent of combat experience may later act as triggers for a soldier with PTSD, but some cues may act as more potent triggers than others. For instance, a helicopter flying nearby at extremely low altitude may be more likely to initiate PTSD symptoms than observing a helicopter flying overhead at high altitude. Such asymmetries in trigger intensity also have a role in widely used treatments for PTSD, including prolonged exposure and cognitive behavioral therapies. Exposure early in the treatment course (for example, imaginal exposure) uses low-intensity triggers for the patient to process through techniques such as corrective learning. Early treatment success then leads to titrating exposure to high-intensity triggers (for example, *in vivo*).^56^ Intensity-based fear generalization is thus a novel technique to examine fear generalization in trauma and other stress-related disorders, and may provide additional insights into the neurocircuitry of PTSD that complements perceptual similarity-based techniques.^57, 58^

### Limitations and strengths

Although exclusion of participants taking psychotropic medication has been the accepted orthodoxy, leaders in PTSD neuroimaging have argued for their inclusion.^59^ Moreover, we found no significant association between generalization bias in fear neurocircuitry and depressive symptoms, alcohol use and common psychotropic medications.^30, 60^ Our PTSD group had greater depressive symptoms than the trauma-exposed control group, but a PTSD group without depressive symptoms has limited clinical relevance, and new evidence calls into question whether PTSD and depression are distinct entities among trauma-exposed individuals.^61^ Usable skin conductance data were unavailable owing to technical challenges with reliable recording in the MRI environment; future studies should confirm our behavioral findings with psychophysiological measures. Our sample consisted mostly of male veterans from the Iraq and Afghanistan conflicts, urging caution when generalizing these results to other demographic groups.

Our design had two generalized stimuli of greater intensity than CS+, but only one generalized stimulus of lower intensity than the CS+. An additional stimulus with the lowest intensity served as the CS−. This design feature, which oversamples generalization toward the higher end of the spectrum, is a minor concern, given that the same design was used for both the groups and did not differentially affect our ability to detect generalization to higher or lower intensity stimuli in a particular group (Supplementary Figure 1; Supplementary Table 2). Moreover, our earlier work^8^ indicates that generalization in this paradigm is asymmetric and is driven by threat intensity present in S4 and S5. That is, a group for whom the S5 served as the CS− did not show a reverse gradient skewed towards the S1.^8^ To fully clarify fear generalization in PTSD, future studies should incorporate other intensity dimensions (for example, loudness and brightness) as well as non-intensity dimensions (for example, shape or size). Further research will also help relate findings in PTSD patients to forms of fear generalization found in other anxiety disorders.^15, 16^

Finally, we frame fear generalization results in PTSD in terms of an associative learning mechanism, such that shocks predicted selectively by the CS+ induce asymmetric intensity-based gradients. An alternative, nonassociative account is that shocks alone are sufficient to induce generalization (that is, sensitization).^57^ Given that our analyses used a CS− as a nonassociative control stimulus and we found evidence for asymmetric intensity-based generalization gradients in shock expectancy ratings in many of the brain ROIs, we rule out a purely nonassociative account of the findings. Another potential interpretation is ‘selective sensitization',^62^ in which mere shock presentations result in sensitivity to high-intensity stimuli selectively.^8^ Given that we also found altered functional connectivity results indicative of impaired safety learning in PTSD, selective sensitization does not fully account for the pattern of results, making associative learning a parsimonious mechanistic interpretation. The relative contribution of associative and nonassociative mechanisms supporting overgeneralization in PTSD merits further theoretical and empirical work.

## Conclusion

Fear neurocircuitry and memory in PTSD are biased toward stimuli that possess greater emotional intensity than the original conditioned-fear stimulus. This study contributes to a growing appreciation that fear-conditioning processes in PTSD^63^ are subject to modifications that take place beyond the initial fear learning episode to make fear memories more resistant to extinction, less contextually specific, and overgeneralized. These functional brain changes may contribute to symptoms of PTSD, which are frequently triggered by trauma cues that merely resemble, but are not identical to cues in the index trauma.

## Figures and Tables

**Table 1:** GLM results of fear generalization for regions of interest

*Region of interest*	*Main effect of diagnosis*	*Fear-level × diagnosis*	*Main effect of fear-level*	*Main effect of time*	*Anti-depr*	*AntiΨ*	*Benzo*	*Mood stab.*	*AUDIT*	*CES*	*BDI*	*CTQ*
Amygdala-R	F_1,56_=18.4; *P*<0.0001	F_1,56_=0.53; *P*=0.59	F_1,56_=0.08; *P*=0.92	F_1,56_=0.58; *P*=0.45	0.33	0.97	0.76	0.85	0.51	0.46	0.19	0.55
Calcarine-R	F_1,56_=4.99; *P*=0.03	F_1,56_=3.17; *P*=0.05	F_1,56_=5.23; *P*=0.01	F_1,56_=0.03; *P*=0.87	0.08	0.41	0.85	0.16	0.23	0.03	0.69	0.02
Fusiform-R	F_1,56_=2.72; *P*=0.10	F_1,56_=3.96; *P*=0.02	F_1,56_=1.92; *P*=0.16	F_1,56_=0.30; *P*=0.59	0.04	0.62	0.36	0.96	0.22	0.09	0.94	0.20
IFG-R	F_1,56_=4.42; *P*=0.04	F_1,56_=2.79; *P*=0.07	F_1,56_=4.78; *P*=0.01	F_1,56_=3.59; *P*=0.06	0.51	0.52	0.89	0.61	0.37	0.65	0.82	0.05
Insula-R	F_1,56_=3.06; *P*=0.09	F_1,56_=7.41; *P*=0.001	F_1,56_=4.53; *P*=0.01	F_1,56_=7.28; *P*=0.01	0.42	0.99	0.81	0.43	0.19	0.68	0.27	0.06
Locus coeruleus	F_1,56_=1.14; *P*=0.29	F_1,56_=4.22; *P*=0.02	F_1,56_=8.4; *P*=0.001	F_1,56_=1.49; *P*=0.23	0.36	0.79	0.80	0.30	0.68	0.002	0.24	0.01
Thalamus-L	F_1,56_=2.65; *P*=0.11	F_1,56_=6.07; *P*<0.005	F_1,56_=4.41; *P*<0.02	F_1,56_=0.84; *P*=0.36	0.15	0.76	0.95	0.46	0.76	0.14	0.40	0.01
Thalamus-R	F_1,56_=3.68; *P*=0.06	F_1,56_=7.38; *P*=0.001	F_1,56_=4.98; *P*=0.01	F_1,56_=0.23; *P*=0.63	0.24	0.81	0.75	0.36	0.88	0.72	0.50	0.01

Abbreviations: IFG, inferior frontal gyrus; L, left; R, right.

Significance levels for covariates indicate interaction of fear-level × covariate for each of: Alcohol Use Disorders Identification Test (AUDIT); Beck Depression Inventory (BDI), Childhood Trauma Questionnaire (CTQ), Combat Exposure Scale (CES), use of antidepressant medication (Anti-depr), antipsychotic medication (AntiΨ), benzodiazepine medication (Benzo) and mood stabilizer medication (Mood stab). Nonsignificant covariates were excluded from the final analysis of variance results reported for each region of interest.

## Supplementary Material

Supplementary Informations
