# Supplementary material for: Fear learning circuitry is biased toward generalization of fear associations in posttraumatic stress disorder
Source: Transl Psychiatry. 2015 Dec 15;5(12):e700–. doi: 10.1038/tp.2015.196 (PMC5068591; doi:10.1038/tp.2015.196)
Supplement: Supplementary Informations [file tp2015196x1.doc]

**SUPPLEMENTARY INFORMATION**

Participants underwent screening for inclusion and exclusion criteria based on information available in our repository and from subsequent telephone contact. Important exclusion criteria included a major Axis I diagnosis (other than depression), contraindication to MRI, substance dependence, traumatic brain injury, and neurological disorders. Structured clinical interviews were conducted with the Clinician-Administered PTSD Scale (CAPS) to ascertain diagnosis and severity of PTSD. Participants completed demographic and clinical questionnaires to assess depressive symptoms (Beck Depression Inventory–II [BDI]), alcohol abuse (Alcohol Use Disorders Identification Test [AUDIT]), drug abuse (Drug Abuse Screening Test [DAST]), childhood trauma exposure (Child Trauma Questionnaire [CTQ]), and current medication use. Upon entry into our repository, participants completed the Combat Exposure Scale (CES) as well as the Structured Clinical Interview (SCID) for DSM-IV to assess comorbid Axis I diagnoses.

*Stimuli and Paradigm*

Stimuli (**Figure 1a**) consisted of a male face morphed along a gradient of emotional expression from neutral-to-fearful taken from the Ekman pictures of facial affect (Ekman and Friesen, 1976). The morphs were positioned in a full-frontal orientation and cropped to remove hair, ears, and neckline. Five morphs were created along the continuum using Morph-Man 2000 software (STOIK) S1 (CS-): 11.11% fear; S2: 33.33% fear; S3 (CS+): 55.55% fear; S4: 77.77% fear; and S5: 100% fear. These face morph values were chosen based on published studies using the same stimuli (Dunsmoor et al., 2009; Dunsmoor et al., 2011) and published psychometric data that individuals can discriminate between specific morph increments and are perceptually sensitive to morphed feature changes that are more subtle than morphs in the present study (Graham et al., 2007; Thomas et al., 2007). The US consisted of an electrical stimulus of 6-ms duration delivered to the right wrist with a voltage level (20-100 V-DC) that was custom calibrated for each participant using an ascending staircase procedure to be deemed “highly annoying, but not painful” (Dunsmoor et al., 2009; Dunsmoor et al., 2011).

The experimental paradigm was based on Dunsmoor et al. (2011), (**Figure 1b**). The experiment began with a short habituation phase that included 1 presentation of each of the 5 morph increments, which allowed participants to get accustomed to the experiment and to reduce orienting responses. Data from habituation were not analyzed. The scanning session consisted of three consecutive stages that occurred in the same order for each participant: preconditioning (3 runs), fear conditioning (2 runs), and generalization (4 runs).

*Preconditioning stage*

Preconditioning contained a total of 12 trials of each of the 5 morph increments (60 total trials) with an average inter-trial interval (ITI) of 6.5 s (jittered across trials according to an exponential distribution function in a range from 5 s to 8 s). For this baseline pre-conditioning stage, the stimulus duration of each face was 4 s, during which time subjects were asked to rate the level of fear expressed by the facial morphs using a joystick on a visual analog scale displayed directly beneath the face. The ITI consisted of a white fixation cross on a black background that followed the offset of each trial.

*Fear conditioning stage*

Fear conditioning contained a total of 12 CS- trials and 18 CS+ trials, with 4 s duration and an average ITI of 12.5 s (jittered across trials according to an exponential distribution function in a range of 10 s to 15 s). The CS+ co-terminated with delivery of the US of 6 ms duration on 6 out of 18 trials, whereas the CS− was never paired with the US (partial reinforcement delay conditioning procedure). Partial reinforcement has been used to effectively fear learning in previous studies (Phelps et al., 2004; Schiller et al., 2010). These 6 trials were included as regressors in the fMRI analysis to address the effect of shock on neural activation.

*Generalization stage*

The generalization test contained 8 CS- trials, 12 CS+ trials, and 16 trials of each of the other 3 morph values (68 trials total), with 4 s duration and an average ITI of 12 s (jittered across trials according to an exponential distribution function in a range of 9 s to 15 s). The CS+ was intermittently paired with the US of 6 ms duration on 4 out of 12 trials to offset the effects of extinction over the extended testing session (steady-state generalization test)(Blough, 1975; Honig and Urcuioli, 1981). Subjects were not informed of the CS-US contingencies. For all stages, stimulus presentation was counterbalanced and pseudo-randomized such that no more than two of the same morph increments occurred in a row.

*Expectancy ratings*

For each conditioning and generalization trial, participants were instructed to indicate shock expectancy on a visual analog scale by manipulating a joystick with their dominant hand. The expectancy rating always started for each trial in the middle of the visual analog scale and subjects were permitted to continuously update expectancy responses until the end of the trial, at which time the position of the joystick position was recorded as the expectancy rating. Thus, no reaction time or motor response information was recorded.

*Post-generalization memory ratings*

At the conclusion of the scan, recognition memory for the CS+ was tested by having subjects select the CS+ from among the five morph values presented on a single montage. Because some participants selected more than one stimulus, a summed score was derived that allocated selection of a single face with two counts and the selection of two consecutive faces (e.g. S4, S5) with a single count for each face.

*fMRI acquisition and preprocessing*

Imaging was performed on a General Electric 3-Tesla MR750 scanner. High-resolution structural MRI scans (T1-weighted) were acquired with a 3D-FSPGR sequence containing 1-mm isotropic voxels, TR/TE/flip angle=7.84-ms/2.98-ms/12°, FOV 256-mm^2^, 166 slices, and 1 excitation. Blood oxygenation level-dependent (BOLD) functional images were acquired parallel to the AC-PC line using a SENSE™ spiral-in sequence: acquisition matrix=642; FOV=256-mm^2^; 34 interleaved slices; voxel dimensions=3.75-mm^3^ with no gaps; TR/TE/flip angle=2000-ms/27-ms/60°.

The first four functional images from each run were discarded to account for magnetic equilibration, and remaining images were corrected for head motion using a threshold of 3-mm in any direction. Paradigm timing files were converted to FSL-compatible format and NIFTI data headers were applied to individual subjects’ data. Functional data were preprocessed using FSL version 5.0 [FMRIB Software Library; Oxford University, UK](Smith et al., 2004) that included brain extraction, motion correction, and slice-timing correction, affine registration to the subject’s anatomical image, spatial smoothing with a Gaussian kernel at 5-mm full-width half-maximum, and time series were filtered below 0.11 Hz to eliminate low-frequency drift.

*fMRI data analysis*

Selection of ROIs implicated in generalization of conditioned fear was guided by a hypothesis-generating step that contrasted CS+ > CS- (**Figure 4**), whereas selection of ROIs implicated in generalization of safety signal learning was guided by contrasting CS- > CS+. The contrasts included all subjects (independent of PTSD diagnosis) using whole-brain voxelwise regression with the FSL FMRI Expert Analysis Tool (FEAT). Likewise, the effect of shock on activation was addressed by including a regressor indicating the CS+ trials paired with shock (33% of CS+ trials). Statistical contrasts for CS+ > CS- and CS- > CS+ were corrected for multiple comparisons that were performed on whole-brain data with the FSL two-step cluster-threshold procedure to identify clusters of contiguous voxels with *Z* > 1.96 and then test the significance of resulting cluster(s) at a corrected threshold of *p* < .05 according to Gaussian random field theory (Worsley et al., 1996). The functional ROIs defined by these contrasts were consistent with our prior work in a non-clinical sample (Dunsmoor et al., 2011). Based on this whole-brain correction for multiple-comparison testing at the hypothesis-generating step, subsequent ROI analyses in the hypothesis-testing step did not require a correction for multiple comparisons. Functional ROIs containing voxels that extended into adjacent anatomical regions were masked. For instance, a functionally defined ROI located in the insula that extended to IFG used a mask to omit IFG voxels [Harvard-Oxford Cortical Structural Atlas, Harvard Center for Morphometric Analysis]. The locus coeruleus ROI was based on 8-mm sphere located in the upper pontine tegmentum using prior localization with MRI to visualize neuromelanin pigment (Astafiev et al., 2010). The relatively small size of this structure should be noted in relation to the spatial resolution of fMRI. Consistent with prior research(Dunsmoor et al., 2011), our hypothesis-generating step did not reveal significant amygdala activation with whole-brain multiple comparison correction; thus we used an anatomically-defined amygdala ROI [Harvard-Oxford Subcortical Structural Atlas, Harvard Center for Morphometric Analysis] due to our *a priori* hypothesis regarding the role of the amygdala in fear generalization. While it is reasonable to predict an amygdala response to CS+ vs. CS- during fear conditioning, numerous prior fMRI studies show that the amygdala response habituates quickly within a few trials of fear conditioning (LaBar et al., 1998) and/or is present only on trials with high skin conductance responses (Buchel et al., 1998; Cheng et al., 2003; Dunsmoor et al., 2011). In the present study the CS+ vs. CS- contrast was performed using all the fear conditioning trials and skin conductance responses were not obtained. These factors likely contributed to a negative amygdala result for the overall contrast averaged across all trials. Selection of cortical regions sensitive to images of faces, including the fusiform face area (FFA), was obtained from our prior fear generalization work (Dunsmoor et al., 2011) using a validated face localizer (Morris et al., 2008). Individual subject analyses produced ROI-averaged activation to the face onsets relative to the pre-stimulus baseline for each condition (5 face morph increments), stage (preconditioning, generalization), and image-volume (TR). Data from the generalization stage was extracted for two image-volumes representing the maximal change relative to the pre-onset baseline and contrasted with analogous image-volumes from the same face morph increment during the preconditioning baseline task. This subtraction ensured that results from the generalization test were controlled for individual differences in the baseline response to viewing fear-expressing faces (**Figure S1**). The difference in activation for the facial morphs expressing greater fear (S4, S5) and lesser fear (S2) than the CS+ were compared to the CS- as a control stimulus to assess activation associated with generalization.

Hypothesis testing was conducted in each ROI with a 2 x 3 x 2 repeated-measures design: between-group factor of diagnosis (2-levels;PTSD,control), within-group repeated measure of stimulus-intensity for generalized face-morphs (3-levels; S2 vs. S1, S4 vs. S1, and S5 vs. S1), and a within-group repeated-measure of time (2-levels; image-volumes at 4-s and 6-s post-stimulus onset except amygdala at 8-s and 10-s). Responses to S3 and S1 do not reflect generalization, as the S3 (CS+) and S1 (CS-) were presented during learning. In addition, the response to S3 was not included in the analyses or in **Figure 5** because it continued to be partially reinforced with shock during the generalization stage and therefore would not constitute a fair comparison with non-reinforced face-morphs.

*Amygdala connectivity analysis*

Task-modulated functional connectivity measures were generated for each facial morph using an interaction term between morph-specific onset times as the *task regressor* and the activation time course of the seed region as the *physiological regressor*. The final functional connectivity strengths were baseline-corrected by subtracting preconditioning levels from generalization levels. Mean and maximum connectivity strengths within each target region from the single subject level were input into a full factorial model that included between-group (control, PTSD), and within-group repeated measures for experimental stage (preconditioning, generalization) and intensity of generalization stimuli (S2, S4, S5) as factors. Our analysis of regions involved in fear learning (highlighted in Figure 5) compared activation to the CS- (S1) because it was the non-reinforced condition, which is its corresponding control stimulus. In this case generalization was being assessed with respect to the CS+ (S3). However, the analysis of the vmPFC that we predicted would respond to safety signal learning, compared connectivity to the CS+ because it was the reinforced condition, which represents its corresponding control stimulus. In this case generalization was being assessed with respect to the CS- (S1). Planned comparisons were carried out between PTSD and control groups for each stimulus-intensity.

**Supplementary Table 1. Clinical and Demographic Information by Group**

| **Measure** | **Trauma-Exposed**  **Group (n=35)** | **PTSD Group (n=32)** | **Group Comparison** |
| --- | --- | --- | --- |
| Age, y | 41.89 (11.04) | 42.25 (9.82) | t(65)=.14, *p*=.88 |
| Female sex, No (%) | 10 (28.57) | 6 (18.75) | *Χ^2^*(1)=.89, *p* =.40 |
| Black race ^a^, No (%) | 18 (51.43) | 22 (68.75) | *Χ^2^*(1)=2.6, *p*=.27 |
| Right handed, No (%) | 33 (94.29) | 26 (81.25) | *Χ^2^*(1)=5.28, *p*=.07 |
| CAPS score | 12.11 (14.33) | 86.25 (18.7) | t(65)=18.3, *p*<.0001 |
| CTQ score | 45.00 (13.09) | 58.84 (27.05) | t(65)=2.70, *p*=.01 |
| BDI score | 4.54 (7.79) | 18.78 (12.53) | t(65)=5.64, *p*<.0001 |
| AUDIT score | 2.60 (2.44) | 4.44 (4.24) | t(65)=2.2, *p*=.03 |
| DAST score | 0.51 (1.04) | 1.19 (2.36) | t(65)=1.5, *p*=.13 |
| Maternal education (y post-secondary) | 3.34 (1.37) | 3.13 (1.34) | t(65)=.66, *p*=.51 |
| CES | 5.37 (6.90) | 14.6 (10.79) | t(65)=4.2, *p*<.0001 |
| Serotonergic medication, No, (%) | 1 (2.86) | 15 (46.88) | *Χ^2^*(1)=21.50, *p* =.0001 |
| Antipsychotic medication, No (%) | 0 (0) | 1 (3.13) | *Χ^2^*(1)=.29, *p* =.48 |
| Benzodiazepine medication No (%) | 1 (2.86) | 6 (18.75) | *Χ^2^*(1)=4.51, *p* =.05 |
| Mood stabilizer medication, No (%) | 0 (0) | 5 (15.63) | *Χ^2^*(1)=5.91, *p* =.02 |

Abbreviations: AUDIT=Alcohol Use Disorder Test; BDI=Beck Depression Inventory; CAPS=Clinician Administered PTSD Scale; CES=Combat Exposure Scale; CTQ=Childhood Trauma Questionnaire; DAST=Drug Abuse Screening Test; No=Number; y=years.

^a^ Race information was obtained from subjects based on categories provided by the investigator to confirm its make-up was consistent with the US Military and did not differ significantly between groups.

**Supplementary Table 2. GLM Results of Pre-Conditioning for Regions of Interest**

| **Region of Interest** | **Main Effect of Diagnosis** | **Stimulus-Intensity * Diagnosis** | **Main Effect of Stimulus-Intensity** | **Anti-depr** | **AntiΨ** | **Benzo** | **Mood stab.** | **AUDIT** | **CES** | **BDI** | **CTQ** |
| --- | --- | --- | --- | --- | --- | --- | --- | --- | --- | --- | --- |
| Amygdala-R | F_1,56_=.32; *p* = .57 | F_1,57_=0.02; *p* = .96 | F_1,56_=.02; *p* = .90 | .68 | .11 | .82 | .31 | .35 | .83 | .95 | .96 |
| Calcarine-R | F_1,56_=.38; *p* = .56 | F_1,57_=1.43; *p* = .25 | F_1,56_=.04; *p* = .85 | .58 | .14 | .97 | .90 | .90 | .71 | .79 | .44 |
| Fusiform-R | F_1,56_=.36; *p* = .55 | F_1,57_=10.89; *p* = .002 | F_1,56_=5.17; *p* = .03 | .82 | .68 | .46 | .40 | .11 | .92 | .62 | .92 |
| IFG-R | F_1,56_=1.70; *p* = .20 | F_1,57_=1.95; *p* = .17 | F_1,56_=5.35; *p* = .02 | .23 | .78 | 68 | .40 | .10 | .67 | .26 | .57 |
| Insula-R | F_1,56_=0.01; *p* = .92 | F_1,57_=0.35; *p* = .55 | F_1,56_=.83; *p* = .37 | .77 | .54 | .84 | .62 | .91 | .24 | .62 | .49 |
| Locus Coeruleus | F_1,56_=1.10; *p* = .30 | F_1,57_=0.60; *p* = .44 | F_1,56_=1.98; *p* = .17 | .53 | .31 | .40 | .96 | .96 | .71 | .73 | .80 |
| Thalamus-L | F_1,56_=.03; *p* = .86 | F_1,57_=1.45; *p* = .23 | F_1,56_=3.75; *p* = .06 | .21 | .13 | .96 | .84 | .68 | .11 | .30 | .52 |
| Thalamus-R | F_1,56_=.001; *p* = .99 | F_1,57_=3.68.; *p* = .06 | F_1,56_=4.81; *p* = .03 | .32 | .17 | .78 | .84 | .84 | .23 | .59 | .60 |

.

Significance levels for covariates indicate interaction of fear-level * covariate for each of: Alcohol Use Disorders Identification Test (AUDIT); Beck Depression Inventory (BDI), Childhood Trauma Questionnaire (CTQ), Combat Exposure Scale (CES), use of antidepressant medication (Anti-depr), antipsychotic medication (AntiΨ), benzodiazepine medication (Benzo), and mood stabilizer medication (Mood stab). Non-significant covariates were excluded from the final ANOVA results reported for each ROI

**Supplementary** Table 3. Activation clusters for fear learning and safety learning

|  | **Region of Interest** | **MNI coordinates** | | | **Size ^a^ (voxels)** |
| --- | --- | --- | --- | --- | --- |
|  |  | **x** | **y** | **z** |  |
| fear learning (CS+ > CS-) | ^b^ Amygdala – R | 24 | -4 | -18 | 115 |
|  | Calcarine - R | 10 | -80 | -4 | 931 |
|  | Fusiform – R | 38 | -64 | -22 | 81 |
|  | IFG - R | 40 | 2 | 46 | 658 |
|  | Insula - R | 34 | 20 | -2 | 417 |
|  | Insula - L | -32 | 20 | -8 | 334 |
|  | Locus Coeruleus – B/L | 0 | -32 | -8 | 81 |
|  | Thalamus - L | -2 | -18 | -2 | 318 |
|  | Thalamus - R | 12 | -12 | 0 | 211 |
| safety learning (CS- > CS+) | Precuneus | 0 | -74 | 20 | 72 |
|  | Posterior Cingulate Cortex | -10 | -48 | 30 | 245 |
|  | dmPFC (frontal polar) | 0 | 66 | -2 | 405 |
|  | Superior Parietal - R | 20 | -52 | 56 | 1419 |
|  | Pre/Postcentral Gyrus - R | 38 | -26 | 56 | 843 |
|  | Lingual Gyrus - L | -10 | -74 | -10 | 5361 |
|  | Lateral Occiptal Cortex - R | 22 | -94 | 12 | 470 |
|  | Central Opercular Cortex R | 42 | -12 | 12 | 283 |
|  | Caudate - L | -16 | 18 | 14 | 91 |
|  | Caudate - R | 18 | 20 | 12 | 153 |
|  | Ventromedial PFC | 4 | 38 | -16 | 60 |

^a^voxel size = 3.75mm^3^;

^b^amygdala was an anatomically defined ROI.

Abbreviations: R-Right; L-Left;B/L-bilateral; dmPFC-dorsomedial PFC

**Supplementary Table 4**. Planned comparisons between groups for Fear Levels

| **Region of Interest** | **PTSD vs. Control** | | |
| --- | --- | --- | --- |
|  | **S2 – S1** | **S4 – S1** | **S5 – S1** |
| Amygdala | *t=*2.52; *p=*.01 | *t=*1.82; *p=*.07 | *t=*1.71; *p=*.09 |
| Calcarine | *t=*.50; *p=*.62 | *t=*2.74; *p=*.008 | *t=*2.11; *p=*.04 |
| Fusiform | *t=*.17; *p=*.87 | *t=*2.56; *p=*.01 | *t=*1.83; *p=*.07 |
| Inferior Frontal | *t=*.59; *p=*.55 | *t=*1.30; *p=*.20 | *t=*1.03; *p=*.31 |
| Insula | *t=* - 1.39; *p=*.17 | *t=*1.54; *p=*.13 | *t=*.18; *p=*.86 |
| Locus Coeruleus | *t=*.49; *p=*.63 | *t=*2.06; *p=*.04 | *t=*1.60; *p=*.11 |
| Thalamus | *t=*.03; *p=*.98 | *t=*2.78; *p=*.007 | *t=*1.58; *p=*.12 |
| Subcollosal | *t=*86; *p=*.39 | *t=*.95; *p=*.51 | *t=* - .04; *p=*.97 |

**Supplementary Table 5. Functional Connectivity between Amygdala and Target Regions**

| **Region of Interest** | **Main Effect of Diagnosis** | **Fear-Level x Diagnosis** | **Main Effect of Fear-Level** | **Anti-depr** | **AntiΨ** | **Benzo** | **Mood stab.** | **AUDIT** | **CES** | **BDI** | **CTQ** |
| --- | --- | --- | --- | --- | --- | --- | --- | --- | --- | --- | --- |
| Calcarine | F_1,65_=.87; *p* = .36 | F_1,65_=6.35; *p* = .01 | F_1,65_= 2.79; *p* = .10 | .27 | .77 | .72 | .07 | .56 | .83 | .65 | .41 |
| Thalamus | F_1,65_=.15; *p* = .70 | F_1,65_=3.54; *p* = .06^a^ | F_1,65_=1.97;  *p* = .14 | .56 | .77 | .12 | .20 | .73 | .98 | .28 | .59 |
| Fusiform | F_1,65_=.05; *p* = .83 | F_1,65_=2.05; *p* = .16 | F_1,65_=.69; *p* = .41 | .89 | .27 | .57 | .51 | .60 | .71 | .39 | .60 |
| Ventromedial PFC | F_1,65_=1.16; *p* = .29 | F_1,65_=.4.22; *p* = .04 | F_1,65_=3.36; *p* = .07 | .28 | .17 | .78 | .61 | .72 | .03 | .66 | .50 |

Significance levels for covariates indicate interaction of fear-level X covariate for each of: Alcohol Use Disorders Identification Test (AUDIT); Beck Depression Inventory (BDI), Childhood Trauma Questionnaire (CTQ), Combat Exposure Scale (CES), use of antidepressant medication (Anti-depr), antipsychotic medication (AntiΨ), benzodiazepine medication (Benzo), and mood stabilizer medication (Mood stab). Dependent measure included mean and maximum of connectivity strength within the target ROI that were included as repeated measures. Non-significant covariates were excluded from the final ANOVA results reported for each ROI. ^a^Test of within-subjects effects: F_2,130_=3.37; p = .04

**REFERENCES TO SUPPLEMENTARY INFORMATION**

Astafiev, S.V., Snyder, A.Z., Shulman, G.L., and Corbetta, M. (2010). Comment on "Modafinil Shifts Human Locus Coeruleus to Low-Tonic, High-Phasic Activity During Functional MRI" and "Homeostatic Sleep Pressure and Responses to Sustained Attention in the Suprachiasmatic Area". *Science* **328**.

Blough, D.S. (1975). Steady-State Data and a Quantitative Model of Operant Generalization and Discrimination. *J Exp Psychol* **104**, 3-21.

Buchel, C., Morris, J., Dolan, R.J., and Friston, K.J. (1998). Brain systems mediating aversive conditioning: an event-related fMRI study. *Neuron* **20**, 947-957.

Cheng, D.T., Knight, D.C., Smith, C.N., Stein, E.A., and Helmstetter, F.J. (2003). Functional MRI of human amygdala activity during Pavlovian fear conditioning: stimulus processing versus response expression. *Behavioral Neuroscience* **117**, 3-10.

Dunsmoor, J.E., Mitroff, S.R., and Labar, K.S. (2009). Generalization of conditioned fear along a dimension of increasing fear intensity. *Learning & Memory* **16**, 460-469.

Dunsmoor, J.E., Prince, S.E., Murty, V.P., Kragel, P.A., and LaBar, K.S. (2011). Neurobehavioral mechanisms of human fear generalization. *Neuroimage* **55**, 1878-1888.

Ekman, P., and Friesen, W.V. (1976). Measuring Facial Movement. *Environ Psych Nonver* **1**, 56-75.

Graham, R., Devinsky, O., and LaBar, K.S. (2007). Quantifying deficits in the perception of fear and anger in morphed facial expressions after bilateral amygdala damage. *Neuropsychologia* **45**, 42-54.

Honig, W.K., and Urcuioli, P.J. (1981). The Legacy of Guttman and Kalish (1956) - 25 Years of Research on Stimulus-Generalization. *J Exp Anal Behav* **36**, 405-445.

LaBar, K.S., Gatenby, J.C., Gore, J.C., LeDoux, J.E., and Phelps, E.A. (1998). Human amygdala activation during conditioned fear acquisition and extinction: a mixed-trial fMRI study. *Neuron* **20**, 937-945.

Morris, J.P., Green, S.R., Marion, B., and McCarthy, G. (2008). Guided saccades modulate face- and body-sensitive activation in the occipitotemporal cortex during social perception. *Brain and Cognition* **67**, 254-263.

Phelps, E.A., Delgado, M.R., Nearing, K.I., and LeDoux, J.E. (2004). Extinction learning in humans: Role of the amygdala and vmPFC. *Neuron* **43**, 897-905.

Schiller, D., Monfils, M.H., Raio, C.M., Johnson, D.C., LeDoux, J.E., and Phelps, E.A. (2010). Preventing the return of fear in humans using reconsolidation update mechanisms. *Nature* **463**, 49-U51.

Smith, S.M., Jenkinson, M., Woolrich, M.W., Beckmann, C.F., Behrens, T.E.J., Johansen-Berg, H.*, et al.* (2004). Advances in functional and structural MR image analysis and implementation as FSL. *Neuroimage* **23 Suppl 1**, S208-219.

Thomas, L.A., De Bellis, M.D., Graham, R., and LaBar, K.S. (2007). Development of emotional facial recognition in late childhood and adolescence. *Developmental science* **10**, 547-558.

Worsley, K.J., Marrett, S., Neelin, P., Vandal, A.C., Friston, K.J., and Evans, A.C. (1996). A unified statistical approach for determining significant signals in images of cerebral activation. *Human Brain Mapping* **4**, 58-73.

**SUPPLEMENTARY FIGURE TITLES AND LEGENDS**

**Supplementary Figure 1.** Preconditioning activation and ratings. **(a)** Subjects provided baseline ratings during preconditioning (prior to fear acquisition) of the level of fear expressed by each stimulus (S1 – S5). There was no significant difference in fear ratings between the PTSD and Control groups [*F*_1, 61_=0.01; *p*>.9] nor any stimulus-intensity*diagnosis interaction [*F*_9, 53_=.62; *p*>.6]. As expected there was a strong main effect of stimulus-intensity [*F*_9, 53_=351.25; *p*<.0001]. **(b)** Baseline activation during preconditioning (prior to fear acquisition) in the PTSD and trauma-exposed control groups in response to each of the stimulus intensity levels (S1 – S5) for all regions of interest (locus coeruleus, calcarine, insula, amygdala, inferior frontal gyrus (IFG), fusiform face gyrus (FFG), L-thalamus, and R-thalamus) shows the lack of systematic differences between groups for the high-intensity stimuli (S4, S5) compared to low-intensity stimuli (S1, S2).

**Supplementary Figure 2.** Shock expectancy ratings were significantly higher for the CS+ than the CS- during fear acquisition runs [*F*_1,58_=107.0; *p<*.0001], indicating successful fear learning. There were no difference between groups [*F*_1,58_=.19; *p<*.67] or stimulus-type*group interaction [*F*_1,58_=.002; *p<*.96]. Specifically, there were no between-group differences for S1 [*F*_1,58_=0.03; *p=*.87] or S3 [*F*_1,58_=.08; *p>*.78] during conditioning.

**Supplementary Figure 3.** Results for CS+ > CS- from whole-brain analysis shows activation (Z>1.96; *p*< .05; corrected) in the calcarine cortex (Calc), caudate (Cdt), fusiform gyrus (FFG), inferior frontal gyrus (IFG), insula (Ins), locus coeruleus (LC), middle frontal gyrus (MFG), supramarginal gyrus (SMG), and thalamus (Thal). These regions obtained from the combined groups (n=67) were used as functionally defined regions of interest (ROI) to assess between-group differences by interrogating mean activation within an ROI for each subject.

**Supplementary Figure 4.** The neural learning response in the amygdala was correlation with the CS+ vs. CS- and the S5 activations among the PTSD group (R = 0.37).

**Supplementary Figure 5.** Activation by ROI during generalization for PTSD and trauma-exposed groups without preconditioning baseline correction.

**Supplementary Figure 6.** Activation by ROI during generalization for PTSD and trauma-exposed groups with preconditioning baseline correction.

**Supplementary Figure 7.** Preconditioning activation by ROI relative to S1.
