# Supplementary material for: Fear learning circuitry is biased toward generalization of fear associations in posttraumatic stress disorder
Source: Transl Psychiatry. 2015 Dec 15;5(12):e700–. doi: 10.1038/tp.2015.196 (PMC5068591; doi:10.1038/tp.2015.196)
Supplement: Supplementary Figures [file tp2015196x2.ppt]

## Slide 1
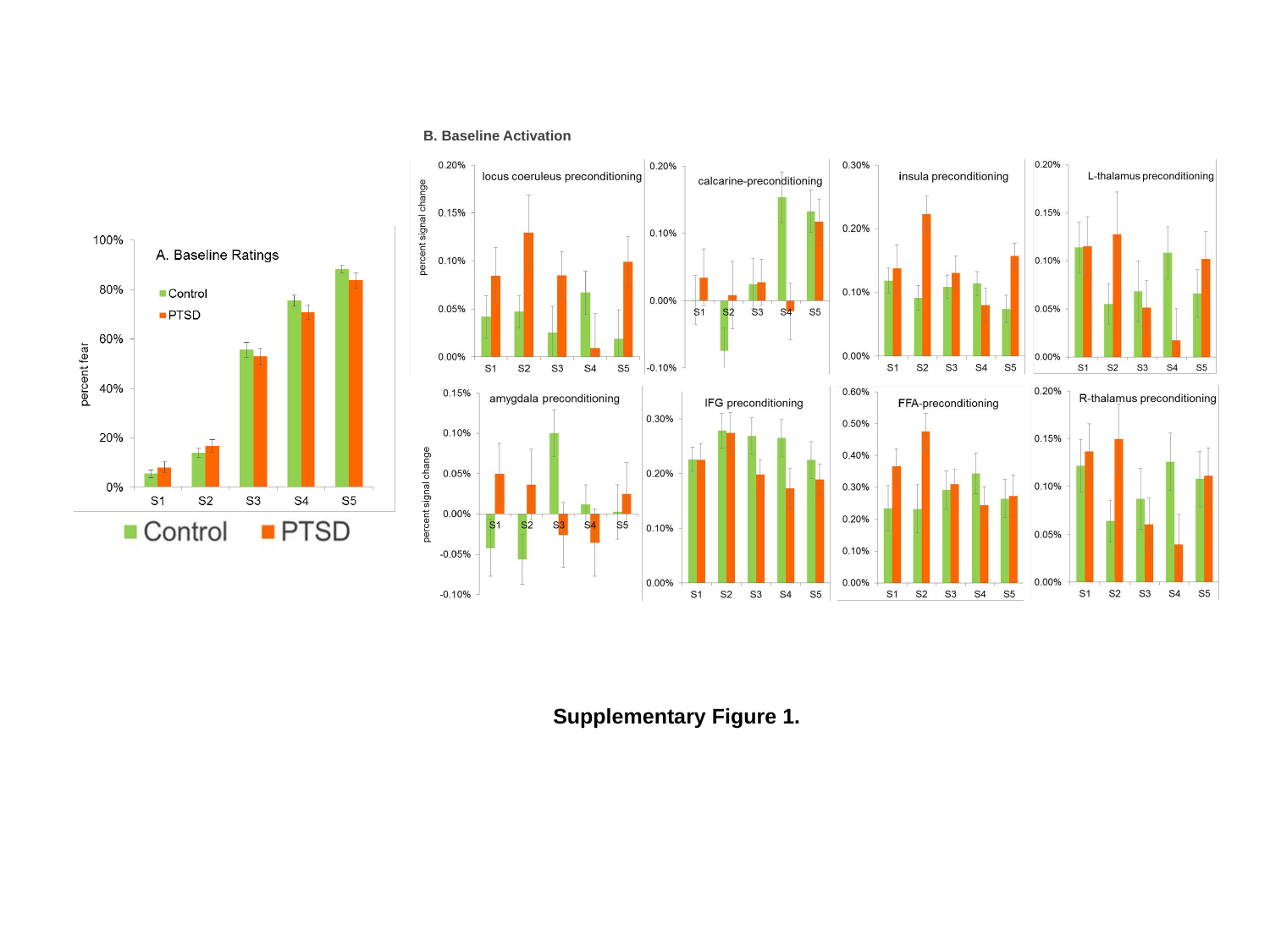

B. Baseline Activation
Supplementary Figure 1.

## Slide 2
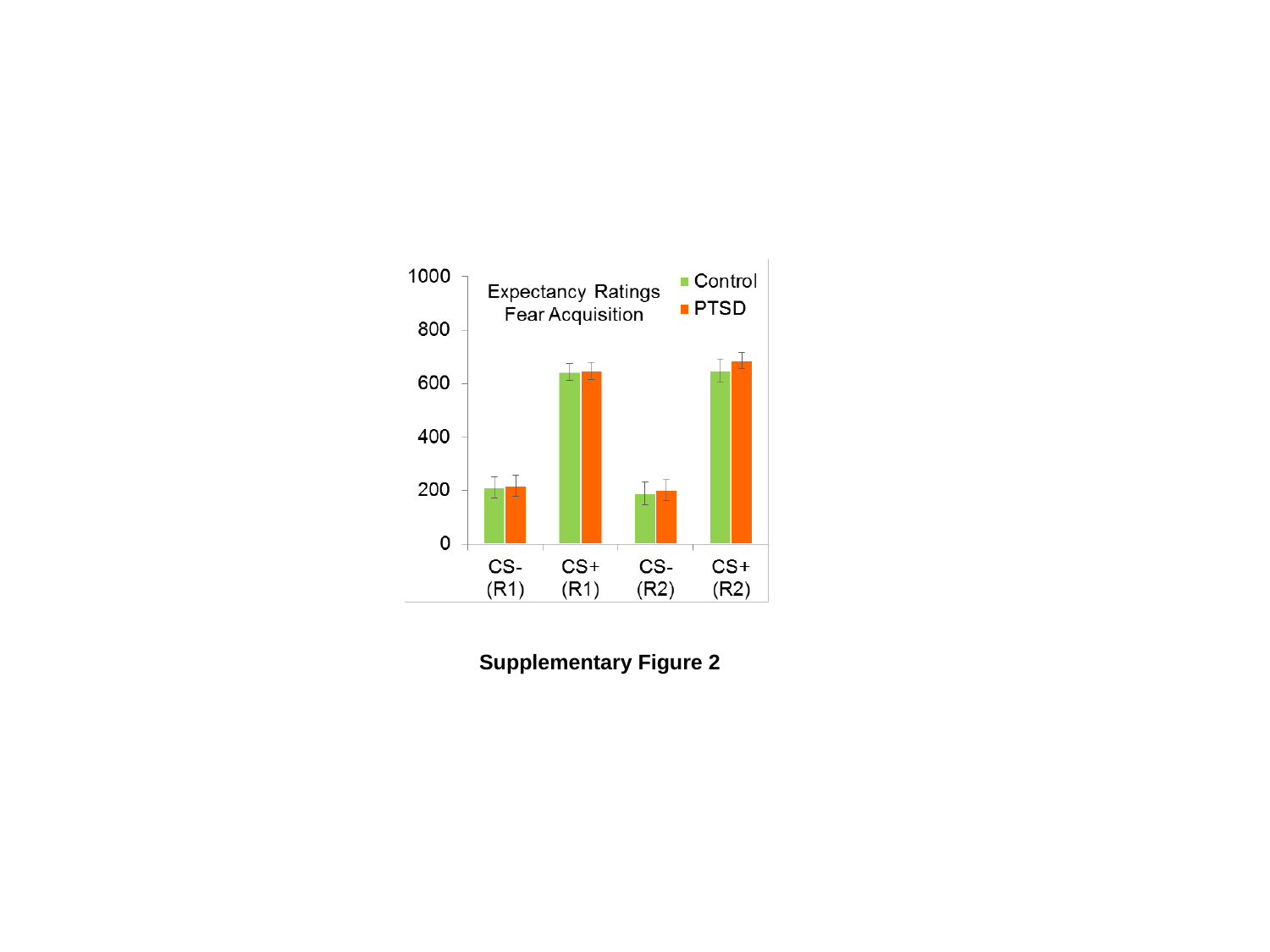

# Supplementary Figure 2

## Slide 3
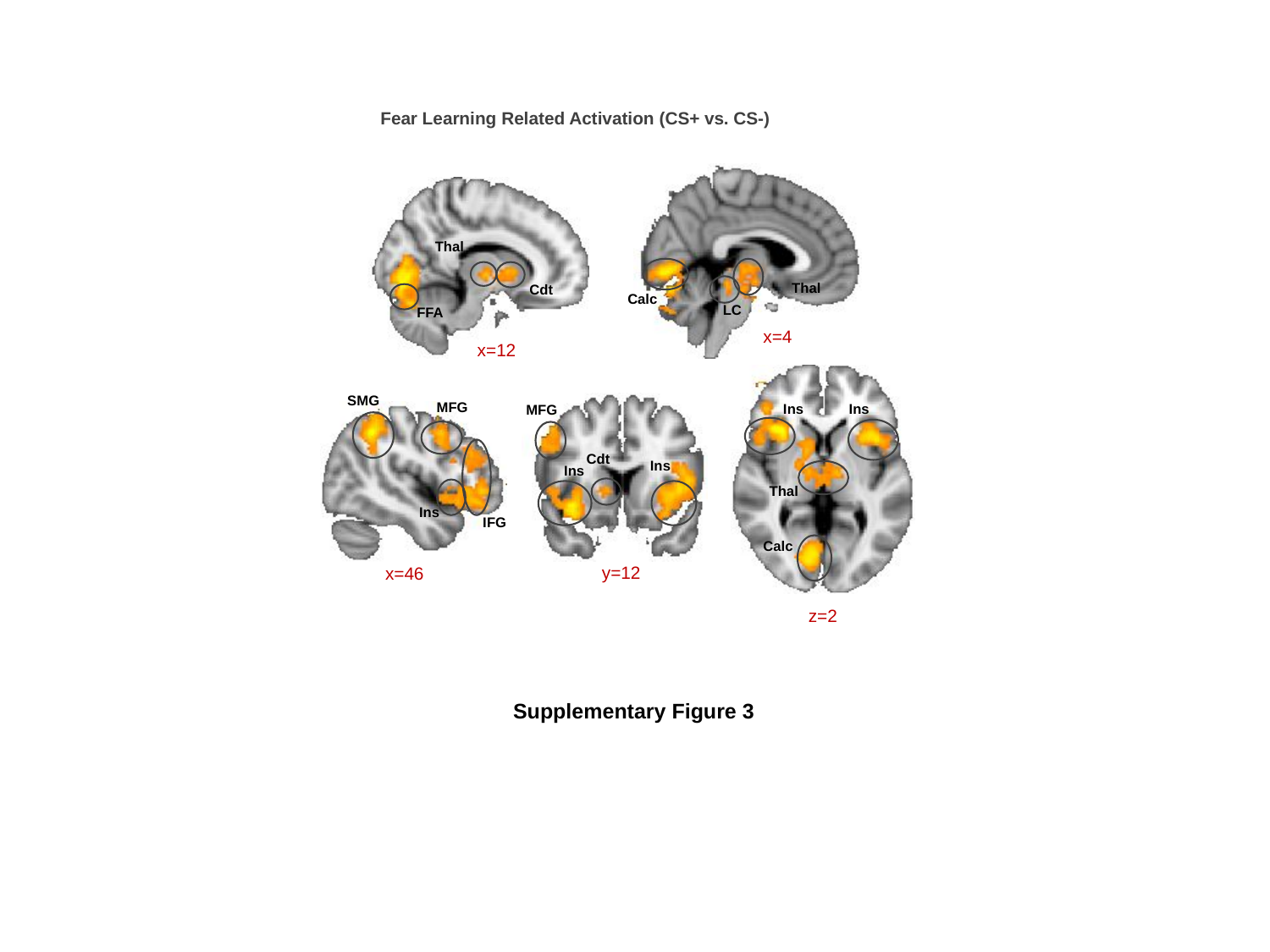

Fear Learning Related Activation (CS+ vs. CS-)
x=4
x=12
Thal
Thal
Cdt
Calc
LC
FFA
z=2
SMG
MFG
y=12
Ins
Ins
MFG
x=46
Cdt
Ins
Ins
Thal
Ins
IFG
Calc
# Supplementary Figure 3

## Slide 4
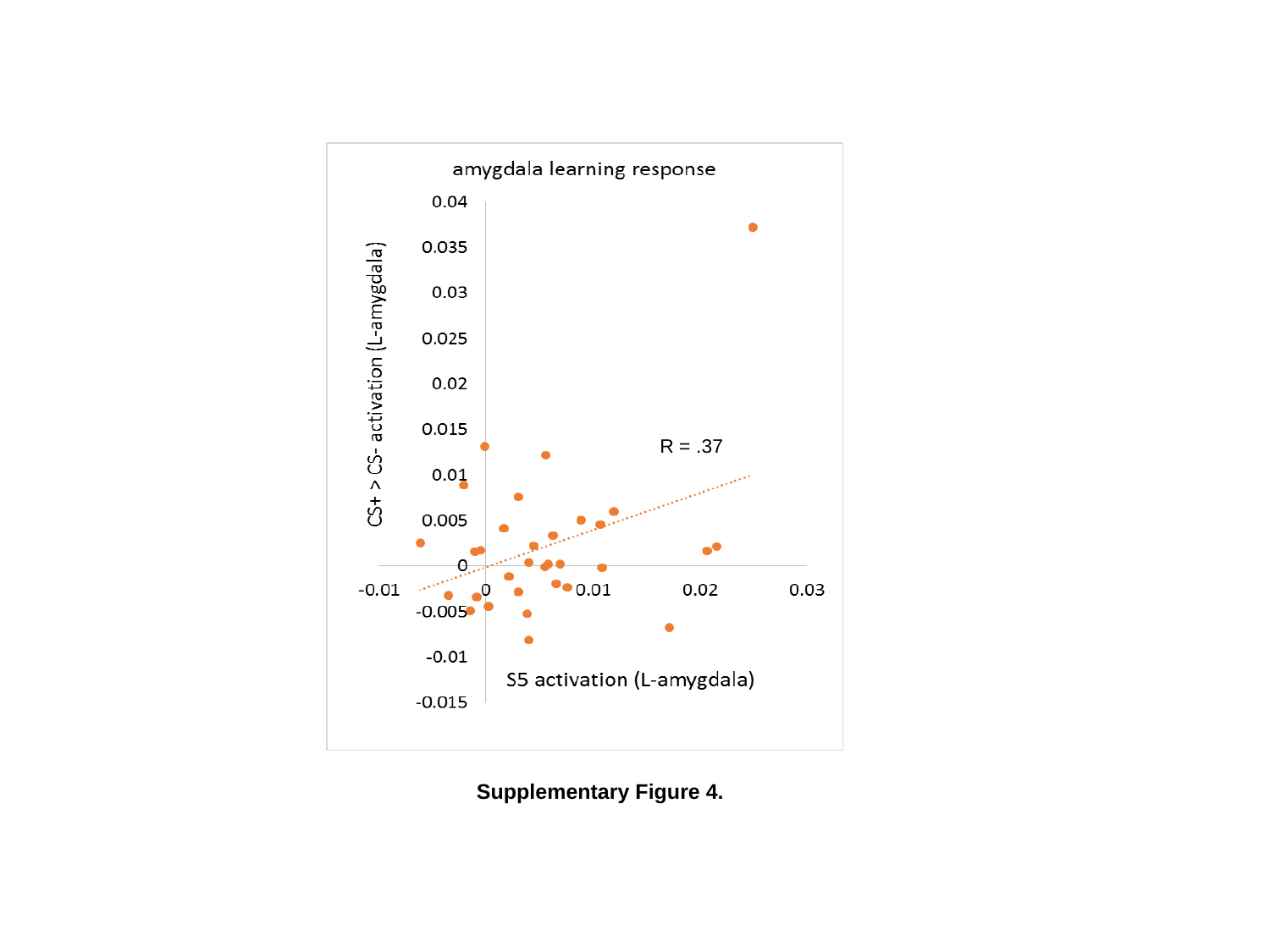

R = .37
Supplementary Figure 4.

## Slide 5
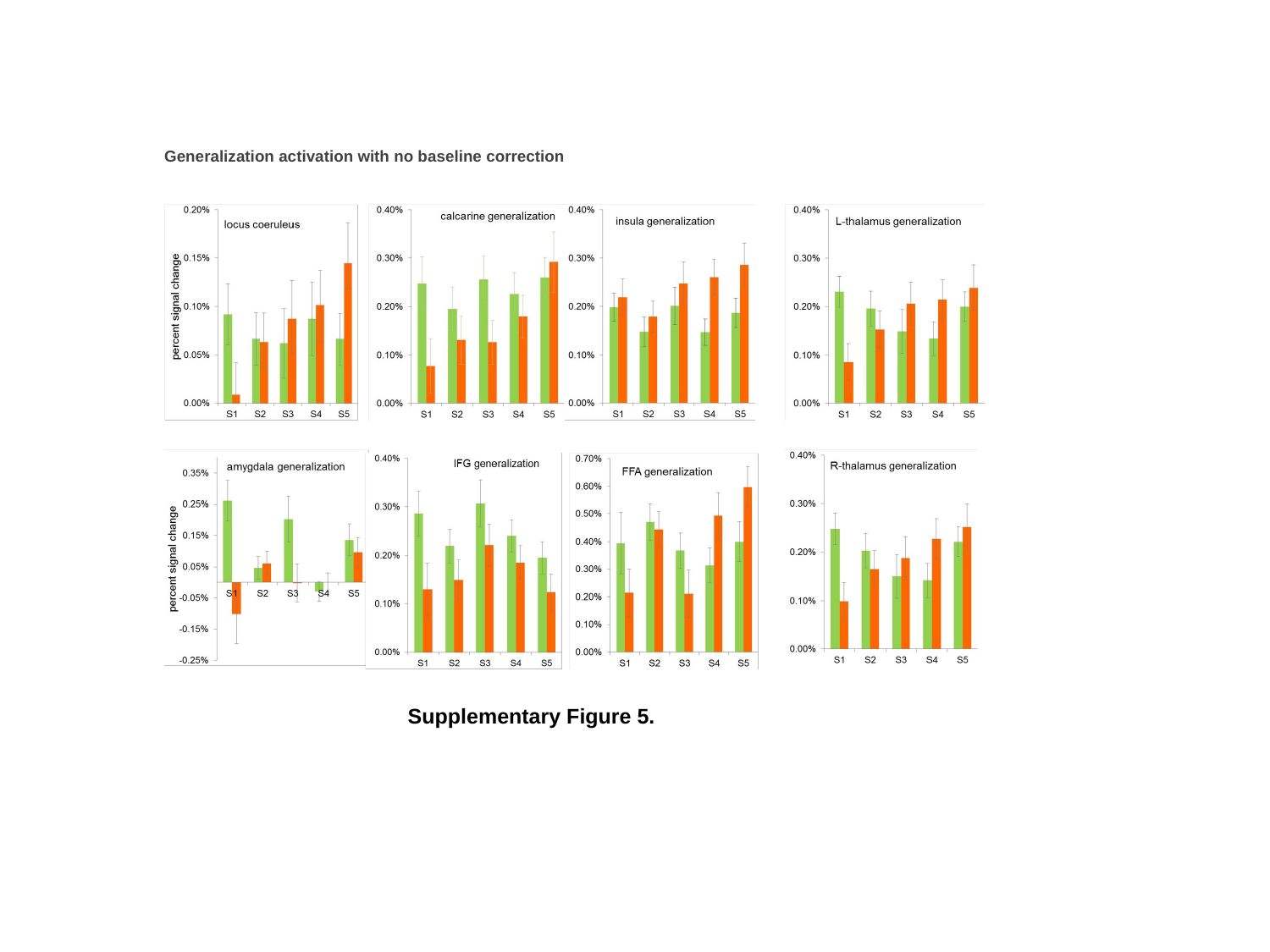

Generalization activation with no baseline correction
Supplementary Figure 5.

## Slide 6
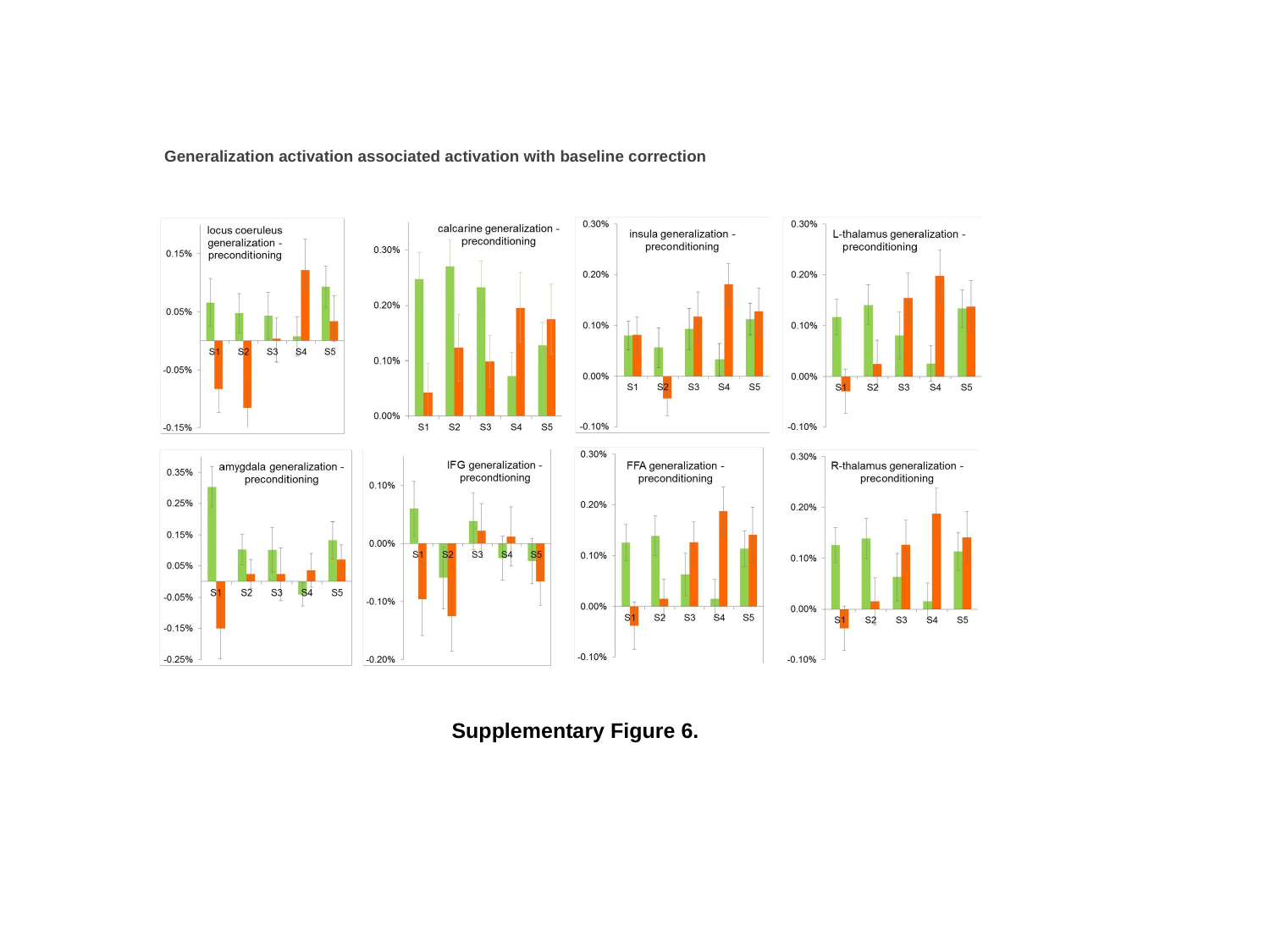

Generalization activation associated activation with baseline correction
Supplementary Figure 6.

## Slide 7
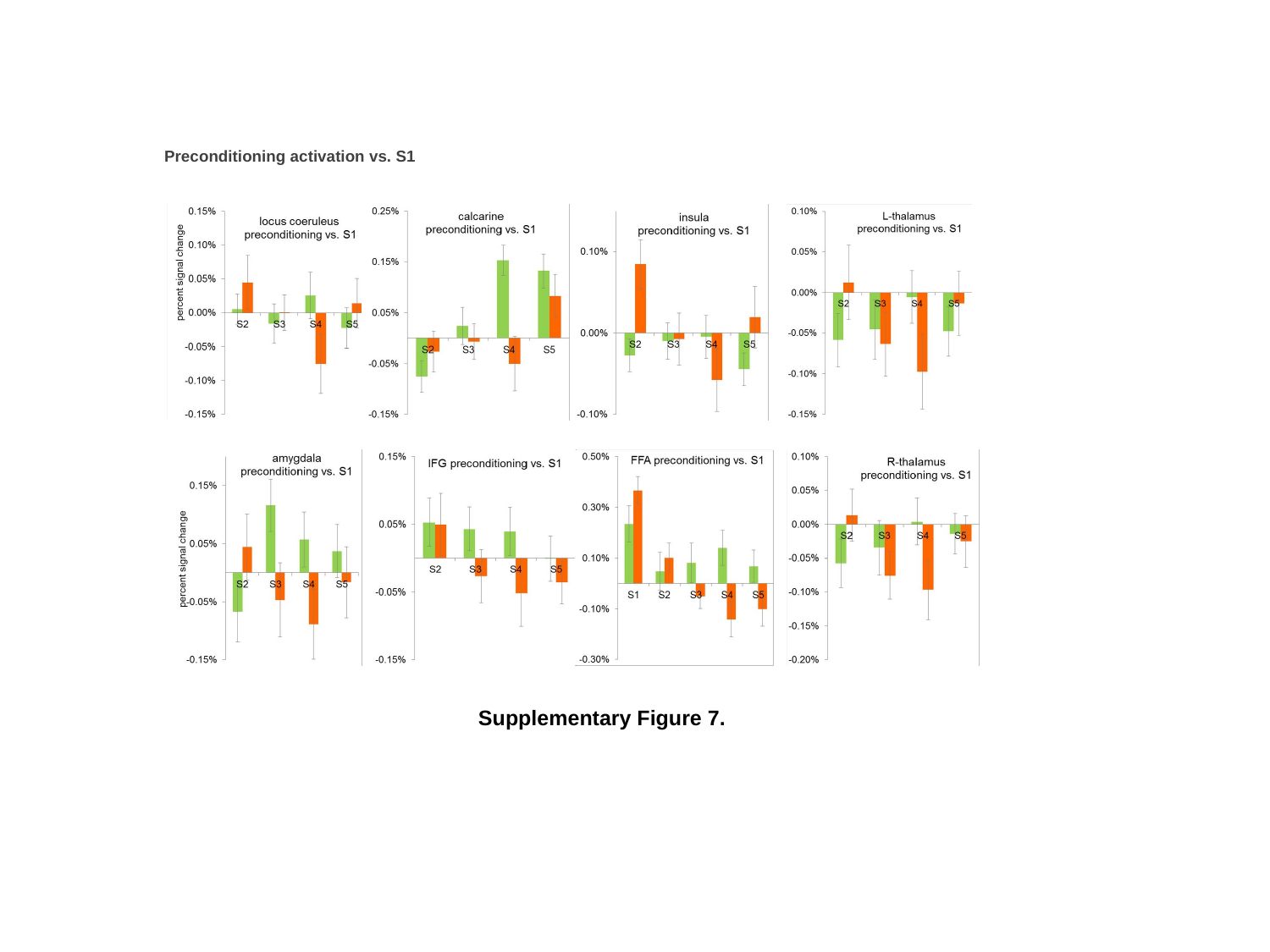

Preconditioning activation vs. S1
Supplementary Figure 7.
